# Energy Performance Assessment of Virtualization Technologies Using Small Environmental Monitoring Sensors

**DOI:** 10.3390/s120506610

**Published:** 2012-05-18

**Authors:** Lu Liu, Osama Masfary, Nick Antonopoulos

**Affiliations:** 1 School of Computing and Mathematics, University of Derby, Derby, Derbyshire, DE22 1GB, UK; E-Mail: n.antonopoulos@derby.ac.uk; 2 B2net Ltd., Dunston Technology Park, Chesterfield, Derbyshire, S41 8NG, UK; E-Mail: omasfary@proact.co.uk

**Keywords:** virtualization, small sensors, Green IT, service oriented computing

## Abstract

The increasing trends of electrical consumption within data centres are a growing concern for business owners as they are quickly becoming a large fraction of the total cost of ownership. Ultra small sensors could be deployed within a data centre to monitor environmental factors to lower the electrical costs and improve the energy efficiency. Since servers and air conditioners represent the top users of electrical power in the data centre, this research sets out to explore methods from each subsystem of the data centre as part of an overall energy efficient solution. In this paper, we investigate the current trends of Green IT awareness and how the deployment of small environmental sensors and Site Infrastructure equipment optimization techniques which can offer a solution to a global issue by reducing carbon emissions.

## 1. Introduction

Recent years have witnessed the continuing development of the Internet from its original communication purpose (e.g., email) and content provision (e.g., Web) to an application deployment platform, where increased computing and storage capabilities are constantly being made available to end users. In parallel, an unprecedented number of personal computers are deployed worldwide according to a recent Gartner report, as worldwide PC shipments have reached 82.9 million units just in the second quarter of 2010, representing a 20.7% increase from the second quarter of 2009. At the same, however, enormous energy has been wasted due to idle resources. A recent report from the NRDC [1] similarly confirmed that most idle servers consume approximately 69–97% of the total power consumption when they are fully loaded, and often when power management function is enabled. With energy costs increasing as the size of IT infrastructures continue to grow, it is apparent that keeping the running costs down is quickly becoming a top priority for many IT centric organisations. In this paper, we will address how to integrate environmental monitoring sensors and the cutting-edge virtualisation technologies to cut power consumption of IT infrastructure.

Recently, cloud computing paradigm has emerged as an energy efficient approach which enables ubiquitous, on-demand network accesses to a shared pool of flexibly reconfigurable computing resources including networks, servers, storage, applications, and services that can be rapidly deployed with minimal management effort or service provider interactions. In particular, so called virtualisation-based cloud computing platforms are becoming very popular in providing a new supplement, consumption, and delivery model for network software application (*NetApp*) over the Internet. Here, virtualisation refers to the abstraction of computer resources, such as the process of running two or more operating systems on a single set of physical hardware.

Originally developed for the IBM mainframe operating systems in the 1960s, the virtualisation technology enables a system administrator to combine disparate physical computing systems into virtual machines in a maximally energy-efficient manner, thus minimizing idle hardware and hence the overall power consumption. Moreover, virtualization can assist in distributing workload in such a way that servers are either busy, or put in a low power sleep state. This has led to server consolidation, with heightened computer elasticity as well as significantly reduced electricity bills. Based on a software cloud model, a virtualized, scalable and energy-efficient resource management strategy can be developed to facilitate integration of loose-coupled resources, with significantly improved utilisation, and with the added advantage that users can be freed from the often costly administration work including software deployment and maintenance.

With 2% of the world's carbon emissions currently being produced by the IT sector according to a Gartner Press Release [2] and with further estimates to reach 3% by 2020 [3], it is explicable that there have been in depth studies which raise the awareness of data centre energy usage [4]. However, there has been little research on the reduction of power usage and carbon footprint through the deployment of server virtualization technologies and more efficient air flow management methods. This is rather interesting when considering that the cost of data centre electricity costs in the UK has doubled between the years of 2003 and 2007 [5].

The objective of this paper is to investigate the current trends of Green IT awareness and how the deployment of small environment monitoring sensors and Site Infrastructure equipment optimization techniques can offer a solution to a global issue by reducing carbon emissions. In this paper, we (1) use small environment monitoring sensors to explore the implications of air temperature on the power consumption of the IT equipment. (2) explore how server virtualization offers a solution through two categories (Hypervisors and OS) and identify the important factors which define virtualization as a Green technology; (3) investigate the site infrastructure components of the Data Centre using small sensors and how their efficiency could significantly contribute to Green IT; (4) monitor and record the power consumption of physical servers sunder different processing loads; and (5) observe the implication of virtual servers on power consumption under different processing loads.

This rest of paper is organised as follows: Related Work on server virtualization is presented in Section 2. The experiment system design is described in Section 3. The experimental results are analysed and discussed in Section 4. Finally, the conclusion is given in Section 5.

## 2. Related Work

The term hardware virtualization is the process of presenting a set of logical computing resources which could be accessed and shared regardless of geographic location or physical configuration [6]. Although this technology is currently under constant exposure by the media and large organisations as a contributor towards Green IT, it was back in the 1960s when it was first introduced by the IBM Corporation as a method of simultaneous timesharing of mainframe computers [7].

This idea was then further developed to incorporate a hardware abstraction layer or else known as a Virtual Machine Monitor (VMM) which provides interaction between the hardware and software layers [6]. However, Szubert [8] explains that it was not until 1999 when virtualization was adopted by VMware that the concept was finally transferred from being strictly used for mainframes to industry standard 86× hardware. As a result of this, a standard 86× server would then have the capabilities of being partitioned into several virtual machines that use virtualized components. This would then allow the concurrent processing of different Operating Systems and software applications in an independent fashion. Although Panek and Wentworth [9] claim that the ability to run multiple VMs on a single server could reduce hardware costs and IT department overhead, Kappel *et al.* [10] argue that this potentially creates a single point of failure as these VMs are solely depending on the physical server to function correctly.

Goldberg [11] classifies the two types of VMMs as: Type I Hypervisor (OS Level Virtualization) and Type II Hardware Virtualizer (Hypervisor Virtualization).

OS Level Virtualization is considered as one of the common methods for running several independent production VMs on the same physical server [12]. The architecture of this technique uses the Host OS installed under the Virtualization Layer to manage a pool of hardware resources. This architecture is also known as OS sharing as the direct interaction with the hardware resources gives the Host OS the capability of sharing these resources among the VMs. Additionally, research [13] suggests that due to this architecture, greater flexibility is achieved as applications could either run on the Host OS or virtually on the Guest OS. However, Marinescu and Kroger [14] explains that this dependency on the Host OS represents a SPOF which could cause a bottle neck and reduced performance that could be 30% less than a non virtualized environment.

Hypervisor Virtualization is increasingly becoming popular for dedicated servers with a primary purpose of running virtual servers. In contrast to the OS Level Virtualization technique, the Hypervisor Virtualization does not rely on a Host Operating System as its Virtualization Layer directly interacts with hardware resources. With the Virtualization Layer directly connected to the hardware resources, it is able to act similarly to the Host OS within the OS Level Virtualization. This means that the Virtualization layer is able to share resources such as the NIC, CPU, RAM and DISK among the VMs whilst avoiding the unnecessary overhead created by the Host OS [12]. Examples of Hypervisor Virtualization include VMware ESX/ESXi and Microsoft Hyper-V.

The literature has so far identified the different methods which could be applied within a Data Centre (DC) subsystem to promote Green IT. From the research and review, it could be concluded that server virtualization technologies and efficient air flow management could contribute to the Site Infrastructure and IT optimization.

## 3. System Design

Although this is supported by a number of studies which demonstrate the benefits of server virtualization deployment to reduce power consumption [6], there is currently little research which compares the difference in power consumption between different server virtualization architectures. Furthermore, there are also little studies which look into the effects of room temperature on the power consumption of virtualized and non virtualized servers by using environment monitoring sensors.

This study fills the gaps in literature by firstly testing the difference of power consumption between physical, Operation System (OS) level virtualized and Hypervisor virtualized servers under different workloads. Secondly, the experiment is replicated within two different room temperatures to explore how site infrastructure components could affect the power consumption. With the experiment containing a combination of hardware and software components, these will be discussed in the following sections.

### 3.1. Software Components

The software components of the experiment remained constant throughout the entire period of time with the only modified variable being the workload on each server. These software components consisted of Operating Systems, Virtualization Infrastructures and Workload generator.

Firstly, it was decided that the chosen Operating System for the virtual and non virtual machines was Microsoft's Windows XP. Since its emergence in 2004, this operating system's speed, reliability and performance has won it a huge popularity which sets it as the currently most deployed Operating System worldwide [15]. Thus, the use of Windows XP makes it the most suitable choice for the replication of a real world production network. Secondly, VMware technology was chosen for virtualization as it offered a wide range of products such as VMware Workstation and VMware vSphere 4. Additionally, VMware's 80% share of the sever virtualization market reflects its popularity and sets it as the mostly deployed virtualization technology [16]. With the VMware Workstation software chosen to implement OS Level Virtualization, the vSphere 4 Infrastructure holds the components required for Hypervisor Level Virtualization. These components are as follows:

- VMware ESX/ESXi: the virtualization platform for vSphere
- VMware vCenter Server: the central point for the configuration and management of virtualized environments
- VMware vSphere Client: refers to the locally installed client interface to allow users to connect remotely to vCenter Server or ESX/ESXi
- VMware vSphere Web Access: refers to the web interface used for managing virtual machines

Finally, in order to make the virtualized experiment as close to the real world as possible, each server will have to process various tasks over a period of time. Through the use of traffic generation software, a number of workloads could be configured to place the server under different states as illustrated in Table 1.

### 3.2. Hardware Components

The hardware components of this experiment consist of the following:

- **4x Servers:** The servers used in this experiment were carefully selected to match each other's specifications.
- **2x Power Monitor:** The Plug-In Mains Electricity Monitors were used to measure the power consumption of each server.
- **4x Cisco Switches:** These consisted of four Cisco Catalyst 3550 Series Switches with L3 capabilities enabled.
- **1x AKCP SensorProbe 8:** This is a standalone device which works as an environment monitoring device. It contains an 8 Port switch for connecting the AKCP sensors which will collect the temperature of the room.

**Figure 1.**
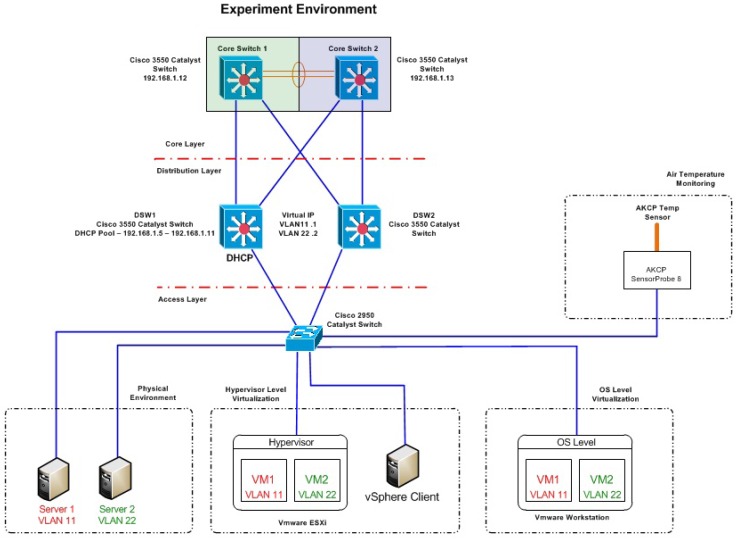
Network topology of experimental environment.

Figure 1 provides a visual representation to the networks hardware and software structure.

### 3.3. Experiment Design

Having identified the software and hardware components required for the testing environment, the OS Level and Hypervisor Level virtualization solutions should be implemented. However, in order to fully simulate a real world environment, the VMs will be configured to automatically obtain an IP address from a DHCP server. This will be required for the testing phase which will also incorporate several network traffic processing workloads.

The experiment will initially compare the two server virtualization architectures to a standard physical architecture. This will examine the potential optimization achieved from IT equipment and in particular hardware virtualization. Furthermore, it is proposed that the experiment will be conducted under two different room air conditions to examine the optimization achieved from Site Infrastructure components such as Computer Room Air Conditioner (CRAC) units. The first room condition is intended to have a high temperature as it is representing a DC server room experiencing from over utilized CRAC units. In contrast, the second room condition of cooler temperature is intended to reflect a DC server room which is being managed by a much more efficient CRAC unit and air flow management techniques.

**Figure 2.**
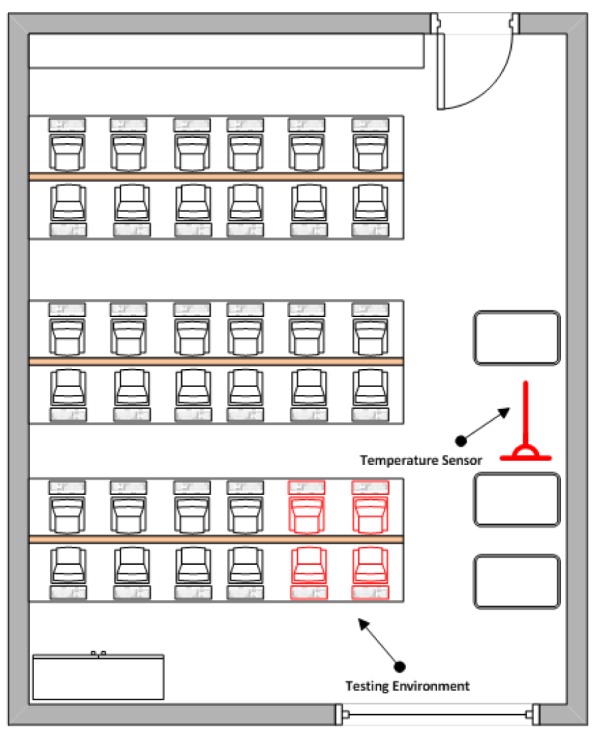
Physical Infrastructure of Testing Environment.

**Figure 3.**
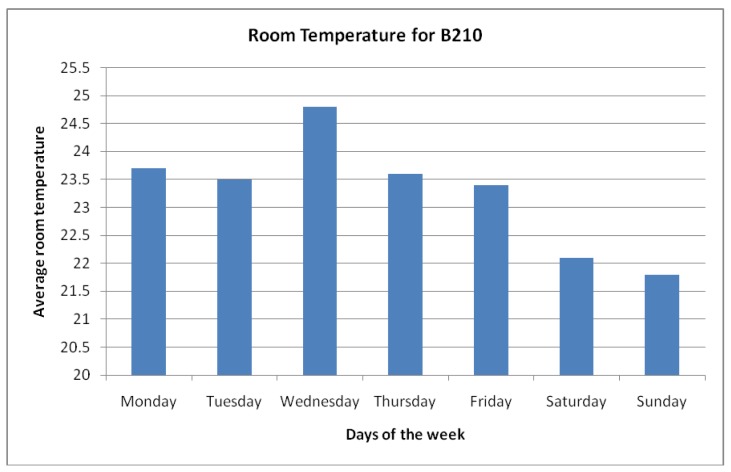
Average Room Temperature measured by environment monitoring sensor.

In order to identify the two periods of the week which reflect the intended room temperatures, a probe sensor was installed within the computer room as seen in Figure 2. By monitoring the air temperature of the computer room for a period of seven days, it was then possible to identify the days with the highest average and lowest average temperatures by using the sensor. This is illustrated in Figure 3 where temperatures are recorded for each day of the week. Through this, it was clear that Wednesday experienced the highest average room temperatures measured by the small sensor. This could be due to a number of factors such as number of students attending classes which resulted in a higher number of computers and network peripherals being used. In contrast, Sunday experienced the lowest average room temperature measured by the small sensors as the room remained mostly vacant due to no teaching schedules.

### 3.4. Primary Data Collection

The primary data of the experiment was collected from a monitoring procedure using power plug monitors and temperature sensors. Firstly, by positioning the power plug in monitors between the AC adapter of the server and the mains power supply, it is then feasible to measure power consumption over periods of time under different workloads. Although there are software based tools for measuring the power consumption, it is understood that the use of physical plug in power meters is cost effective [17] and offers the most accurate method of measuring power of servers running several workloads [18]. Furthermore, by distributing temperature sensors within the computer room, it is then feasible to measure the air temperature over periods of time. The sensors were directly connected to the RJ-45 sensor port switch within the SensorProbe 8 as this is where data will be collected and stored periodically. Finally, with the monitored environment successfully set up, the previously discussed workloads will be performed on each server.

### 3.5. Monitoring

This phase of the experimental procedure encompasses the rotation of processing workloads and air conditions to measure the impact on the power consumption. The experiment compromised two environments (Cool, Warm), with three devices monitored under three workload phases for a period of 90 seconds. This was designed to enable any trends in power consumption to be detected.

### 3.6. Replications

In order to verify the accuracy of the measured data, it is critical to ensure that a fair monitoring procedure is being carried out. Thus, it is proposed that test replications will be carried out as they offer increased confidence in the accuracy of the produced results [19]. Basili *et al.* [20] cateogrizes replications into different types as illustrated below:

- Replication that do not alter the hypothesis.
- Replication that alter the hypothesis.
- Replications that reformulate the goals of the experiment.

For the purpose of this experiment, it has been decided that two types of replications will be adopted. Firstly, the Replications that do not alter the hypothesis will be conducted for the purpose of verifying the accuracy of the results. This includes the repetition of the original experiment 3 times as closely as possible without any alterations to the other variables. Secondly, the *Replications that alter the hypothesis* will be based on the alteration of air conditions to verify the impact of room temperature/ computer processing workloads on the power consumption.

## 4. Results and Discussion

The results of the power consumption were split into two sections; cool air temperature data and warm air temperature data. This was designed with the intent of exploring any similar trends within each environment. In addition, this would allow the comparison of results between the environments to clearly identify any effects experienced by the variant air temperature.

### 4.1. Cool Air Temperature Data

**Figure 4.**
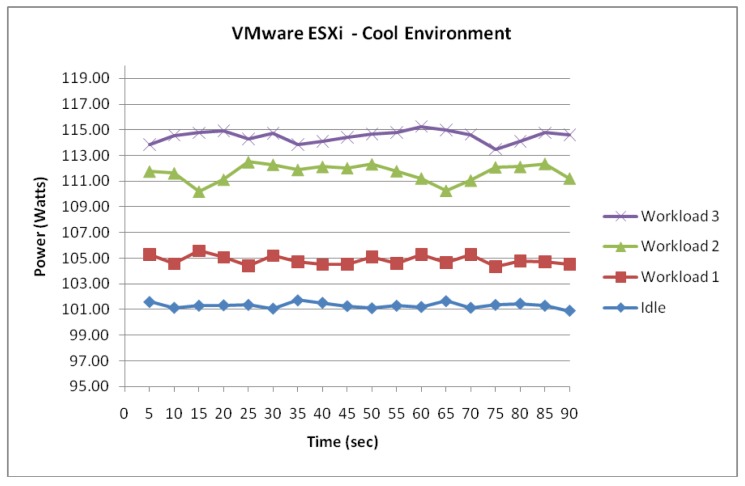
ESXi server functioning within a cool environment.

**Figure 5.**
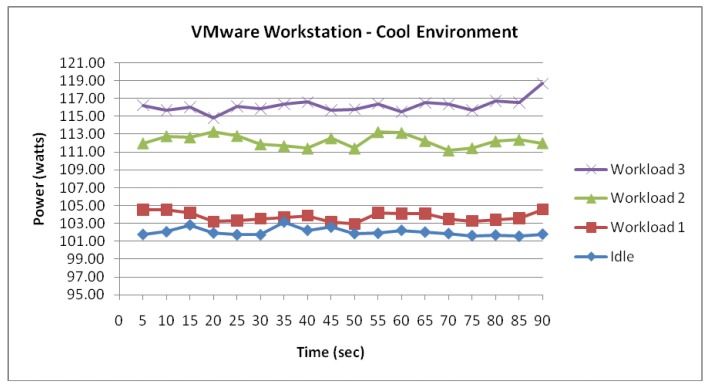
Workstation functioning within a cool environment.

**Figure 6.**
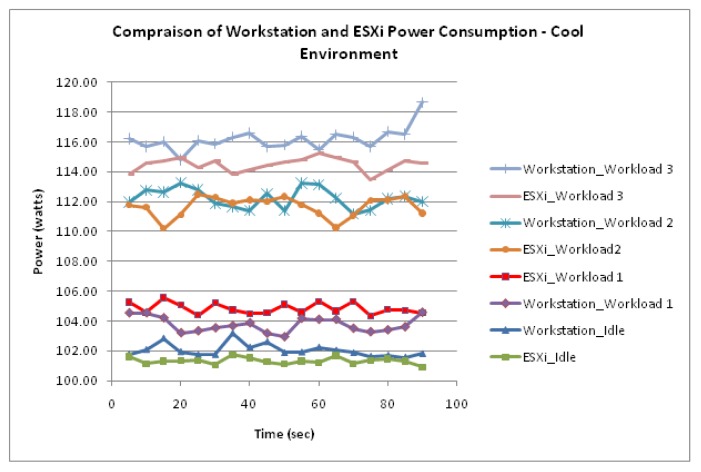
Difference in power consumption between Workstation and ESXi.

**Figure 7.**
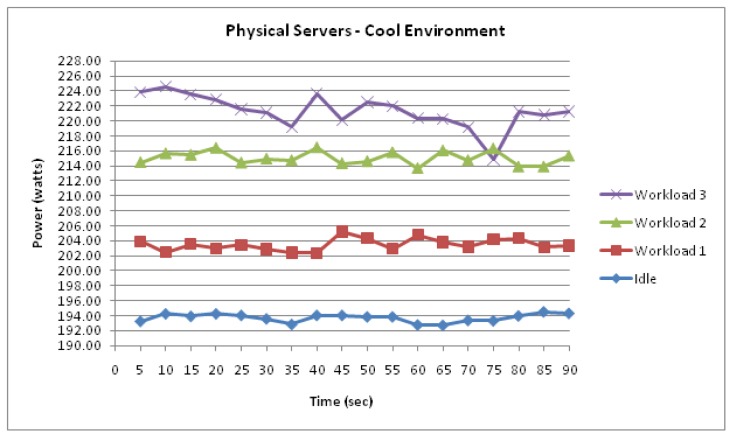
Physical Server functioning within a cool environment.

**Figure 8.**
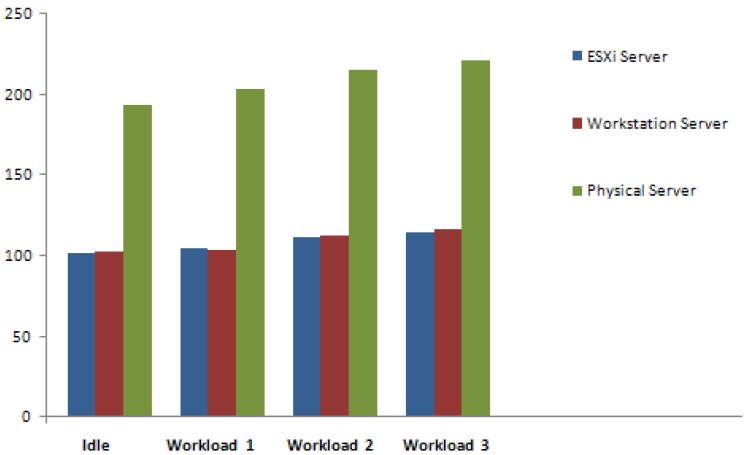
Difference in power consumption between physical and virtualized servers.

Figures 4–8 show conclusively that each server is drawing an increasing amount of electrical power whilst under significant processing workloads. In addition, it has been observed that the recorded data for each server whilst idle has experienced the most stability and lowest range of fluctuation. In comparison, the highest magnitude of fluctuation was observed once each server has been configured with workload 3.

#### 4.1.1. ESXi

The ESXi server running two VMs has experienced an incremental power response to the 3 workloads as illustrated in Figure 4. Whilst idle, the power consumption of the server remaining consistent averaging, 101.3 Watts with a fluctuation range of 0.6.

The measured data shows that once the system was configured with processing workloads equaling to 10–15% Utilization rate (Workload 1), the power consumption increased more than 3.5 Watts. With the power intake now averaging 104.8 Watts, you could see from Figure 4 that the values are still following a similar pattern from the previous configuration but with a higher fluctuation equalling 1.23 Watts.

This trend in incremental power consumption was further evident as the server experienced higher utilization rates. The measured data suggest that the highest increase in power consumption occurred as the server transited from Workload 1 to Workload 2. This was measured at a 6.83 Watts increase as the server processing Workload 2 averaged 111.6 Watts over the 90 second period. Furthermore, measured data suggests that once the server was configured with processing tasks equalling utilization rates of 25–30% (Workload 3), the measured data showed a slight increase of 2.83 Watts by now averaging 114.5 Watts.

#### 4.1.2. Workstation

Figure 5 shows that the Workstation server running two VMs has also experienced an incremental power response to the three workloads. In addition, Figure 5 suggests that the Workstation server representing OS level virtualization has experienced similar power consumption values to the ESXi which represents the Hypervisor level virtualization.

Figure 6 visualizes the similarity of consumption between the two server virtualization technologies as each workload was configured. Firstly, whilst idle, the measured data shows that the power consumption of the Workstation server sustained stability averaging 102 Watts, with a small fluctuation of 0.67. When the Workstation data was compared to the measured data for the ESXi server, a difference of 0.7 Watts was evident and thus proving a strong degree of similarities in consumption between the two technologies.

Moreover, the trend in similarity between Workstation and ESXi was also found as the Workstation server was configured with workloads 1, 2 and 3. Workload 1 for the Workstation server displayed a power consumption averaging 103.7 Watts. When compared to the power consumption of Workload 1 for the ESXi server a difference of 1 Watt was observed. Workload 2 for the Workstation server displayed a power consumption averaging 112.2 Watts. When compared to the power consumption of Workload 2 for the ESXi server a difference of 0.5 Watts was observed. Furthermore, Workload 3 for the Workstation server displayed a power consumption averaging 116.1 Watts. When compared to the power consumption of Workload 3 for the ESXi server, a difference of 1.7 Watts was observed.

#### 4.1.3. Physical Server

Figure 7 visualises the two physical servers' combined incremental power response to the 3 workloads. Again, similarly, the measurement data shown in Figure 7 demonstrate another strong correlation between the power consumption and workload.

The measured data also suggest that the physical servers experienced further similarities. Firstly, it was observed that the measured consumption whilst idle experienced the most stability. Moreover, the results also suggest that the highest increase in consumption occurred during the transition from workload 1 to workload 2. It is notable that the scale of consumption is the most apparent difference between the physical servers and previously discussed server virtualization technologies. This is illustrated in Figure 8 where the difference in power consumption between the physical and virtualized servers is demonstrated. This energy inefficiency of physical servers is evident as they are almost doubling the power intake of virtualized servers.

### 4.2. Warm Environment Data

**Figure 9.**
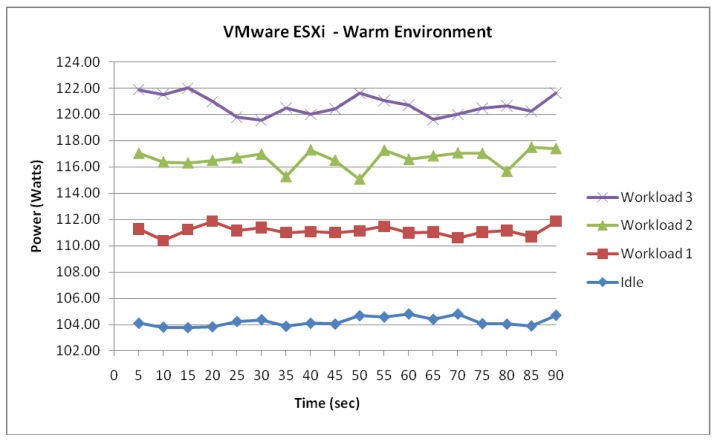
ESXi Server functioning within a warm environment.

**Figure 10.**
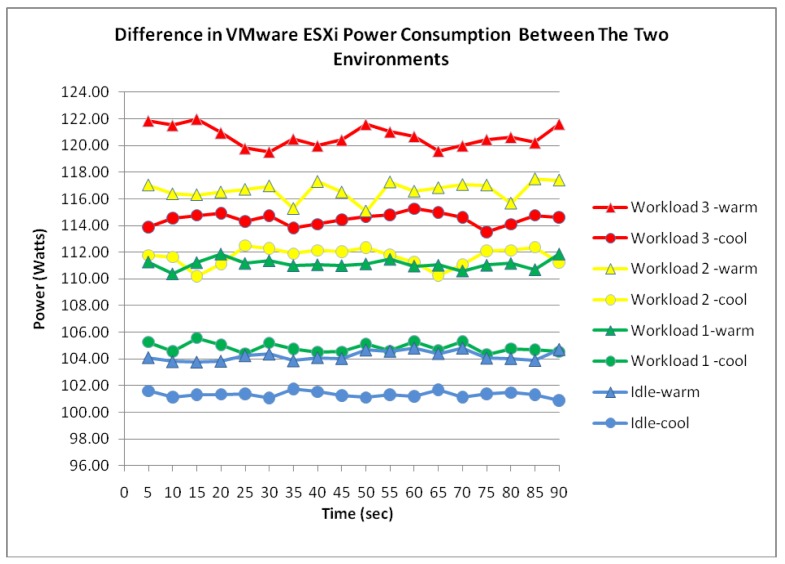
ESXi power consumption difference.

**Figure 11.**
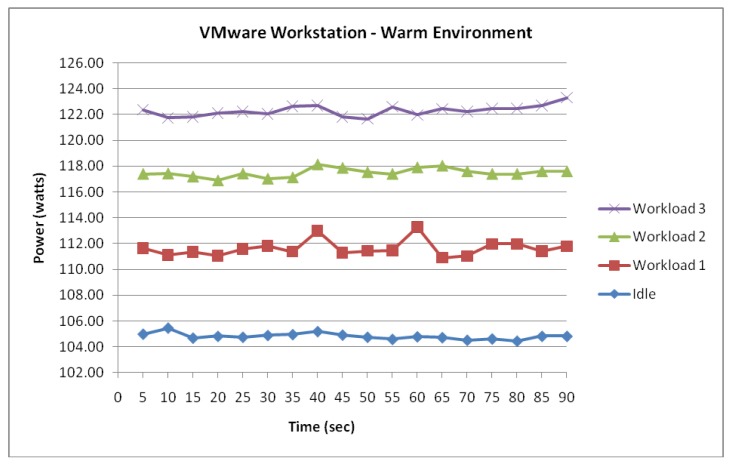
Workstation Server functioning within a warm environment.

**Figure 12.**
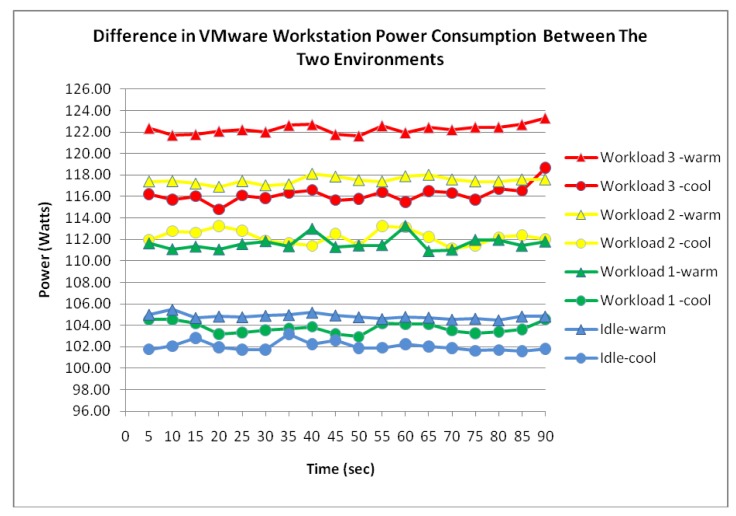
Workstation power consumption difference.

**Figure 13.**
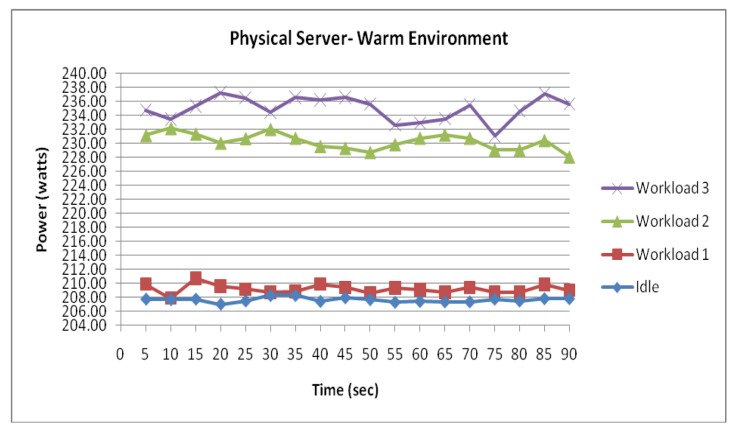
Difference in power consumption between Workstation and ESXi.

**Figure 14.**
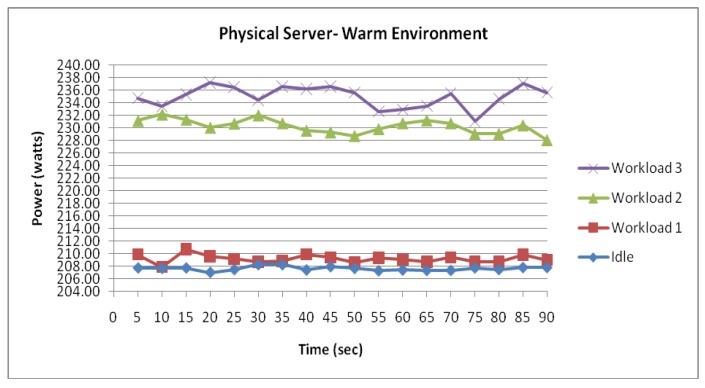
Physical Server functioning within a warm environment.

**Figure 15.**
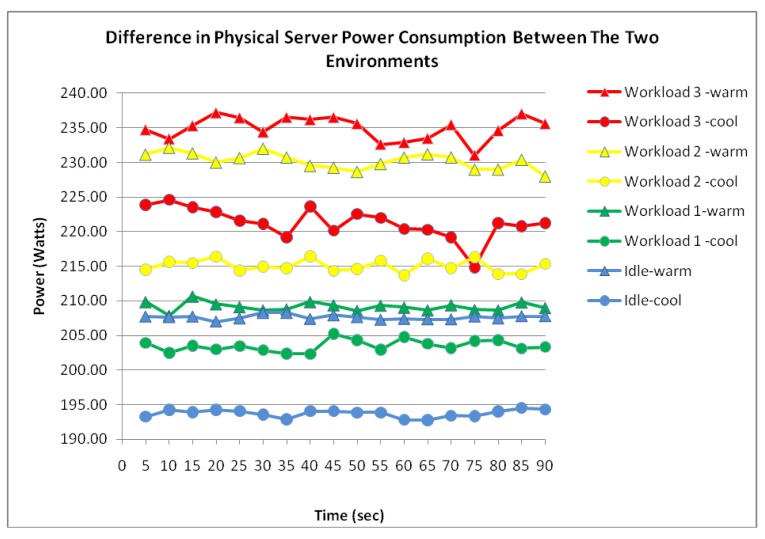
Physical server power consumption difference.

Figures 9–15 visualize the measured power draw of each server functioning within a warmer air environment than the previous set of data. Again, it is notable that each server is drawing an increasing amount of electrical power whilst under significant processing workloads. Similarly to Figures 4–8, it was observed that the power consumption for each server whilst idle experienced the most stability as it recorded the lowest range of fluctuation. However, unlike data shown in Figures 4–8 where the highest increase in power consumption was experienced in the transition from workload 1 to workload 2, this trend was no longer evident within this data set.

#### 4.2.1. ESXi

Figure 9 shows the ESXi server experiencing an incremental power response to the 3 workloads. Whilst idle, it is observed from the measured data that the power consumption of the server remained stable averaging 104.2 Watts. This was suggested from a low fluctuation range of 1.03 Watts. Now, although this stability whilst idle was also evident in the previous environment, a number of differences were observed. When the power consumption of the ESXi server whilst idle is compared to the power consumption from the colder environment, a 2.2 Watts increase in the average consumption was observed. While this may give an indication of the effect of temperature on the power consumption, it was important to identify further trends to support this hypothesis.

After further examination of the remaining data, the existence of correlation between the air temperature and power consumption was further supported as illustrated in Figure 10. It is notable that a similar pattern was highlighted which contains the collected data of workload 1. Although the increase in power consumption was expected due to the configured workload, the server's average consumption of 111.1 Watts experienced a 6.3 Watts increase from the cooler environment. Similarly, the increase in power consumption was evident from the collected measurement data and thus suggesting that the increase in air temperature has certainly affected the level of power consumed by the ESXi server.

#### 4.2.2. Workstation

Figure 11 shows the Workstation server experiencing an incremental power response to the three workloads. Again, it is suggested from the measured data that the server's power consumption is most stable whilst idle. The figure shows the power consumption of the workstation server whilst idle averaging 104.8 Watts with a low fluctuation of 1 Watt. The correlation between the server's workload and power consumption was further evident shown in Figure 11. However, it is notable from Figure 12 that an average increase of 5.5 Watts in power consumption was observed once the data was compared to the measured power consumption from the cooler environment.

Similar to the cooler environment where the power consumption between the two server virtualization technologies experienced very little difference, Figure 13 shows that the measured data of the workstation within the warm environment also suggest power consumption similarities with the ESXi server.

#### 4.2.3. Physical Server

Figure 14 visualises the two physical servers' combined incremental power response to the three workloads. Again, similarly to the physical server within the cool environment, the measured data series demonstrate another strong correlation between the power consumption and workload.

Figure 15 illustrates the main difference between this data set and the previously collected data of the physical server from the cooler environment. Further comparison between the physical server in a warm environment and physical server in a cool environment suggest that the physical server within the warmer environment experienced an average increase of 12.4 Watts in power consumption. With this increase evident throughout the experiment as the workloads were configured, it is clear that the difference in power consumption between the two environments has been largely influenced by the air temperature.

### 4.3. Discussion

This section of the paper will discuss a number of observations which were made upon the completion of the experiment and analysis of the results.

#### Virtualization Technologies and Physical Servers

Firstly, it is clear from the experiment that the use of server virtualization technologies has improved the power efficiency in comparison to the physical servers. Although this data also displays a strong correlation between the power consumption and workload, the results of this experiment are not considered important beyond to what they contribute to the following observation: There is a 103.1 Watts difference between two virtualized servers and two physical servers whilst idle. This observation as seen in Table 2 proves important as it demonstrates the high levels of power being wasted by inactive physical servers waiting for tasks to process. Therefore, by virtualizing underutilized servers into a single physical server, this offers significant power savings as physical servers could be removed. For example, by virtualizing five physical servers running at 15% utilization into five virtual machines within a single physical server, this would potentially eliminate the running costs of four servers.

However, it could be argued that a single server running five virtual machines with a total utilization rate of 75% would require additional processing and therefore consume as much power as the five physical servers. This would not prove the case as Central Processing Units (CPUs) consume approximately ¼ of the server's total power consumption whilst the rest is shared among the other components [18]. This means that the CPU's 60% utilization increase from 15% to 75% only accounts for ¼ of the power and thus, an 18% increase in power consumption is expected from the CPU's additional processing. Furthermore, other components such as the Fan and PSU will also experience an increase in power consumption but this is regarded insignificant when compared to the power that is being saved. This is clearly illustrated from the measured data where the average of two virtualized servers produced a 51.7% savings in power usage from two physical servers processing the same workload.

To demonstrate the potential savings from adopting server virtualization technologies, the following scenario could be assumed from the collected data. By running two underutilized servers at utilization rates of 10–15% (workload 1), an approximate total of 418.2 Watts in power is required. Through server consolidation, the CPU usage of the single server is expected to increase to 25–30% due to the two VMs dependence on shared resources. With the virtualized server running two VMs now averaging 121.4 Watts, the following savings could be achieved:

418.2W−121.4=296.8W

Assuming that the cost of electricity is around 10p per kWh as quoted by British Gas [21], a DC that operates 24 hours a day, 7 days a week, will benefit from server virtualization as follows:
*Watts per hour*:0.2968 kWh*Assuming that the server operates 24/7*:365 days × 24 hours = 8,760 hours*Consumption per year*:8,760 hours × 0.2968 = 2,599.968 kWh*Savings per year*:2,599.968 kWh × £0.10 = £259.99

Furthermore, through the use of server consolidation, a number of additional savings beyond just the electricity cost of the server could be achieved. With Bianchini and Rajamony [22] and the cooling infrastructure, it is critical to understand the implications of inefficient servers on the CRACs. The use of inefficient physical servers that consume more power tends to produce more heat into the server's room. This creates the requirement for a larger and more sophisticated cooling infrastructure to efficiently remove the heated air from the server. Therefore, by consolidating underutilized servers into VMs, the lesser heat produced will reduce the workload on the CRAC units and essentially lower the electrical power required to cool the air. However, with virtualization now reducing the IT workload and produced heat, it is important to avoid the risk of running the cooling infrastructure with more power than required [23]. It is suggested that rightsizing in this scenario should be carefully applied as it will reduce fixed costs and increase efficiency [23].

## 5. Conclusions

Recent advances in sensor technologies and virtualization technologies are providing exciting opportunities to make significant progress in understanding and solving the real-world challenge on reduction of power usage and carbon footprint. This paper has highlighted the emergence of Green IT as a result of the increasing trends in power consumption and discussed a number of measures for efficiency improvements. With these measures divided to either the IT equipment or Site infrastructure subsystem of the DC, it was decided that one of each will be examined for efficiency improvements through direct experimentation. Server virtualization was chosen as part of a solution for IT equipment optimization. In comparison, the efficiency of the CRAC was used as part of a site infrastructure measure for improving the power efficiency of the DC by using small environment sensors.

The measured power consumption obtained from the use of server virtualization technologies appear to save more than half of the power required by physical servers. This can potentially be attributed to the server's primary power consumers being more efficiently utilized through server consolidation. In addition, through the testing of both server virtualization technologies, very similar data was gathered and thus suggesting little difference between the two technologies in the context of power consumption. However, this may not be the same in terms of measuring performance levels as the both technologies have differing virtualization architectures.

Furthermore, it appears that the efficiency of site infrastructure components such as CRACs have a direct effect on the power consumption of IT equipment. This is suggested from the comparison of server power draw between two contrasting air conditions. The first test which was conducted within a warm environment which detected by an environment monitoring sensor recorded the highest power consumption. This could be attributed to the temperature sensitive components of the server. Although the measured data suggest that temperature will affect the overall consumption of the server, it could be concluded that the CRAC units are also expected to experience an increase in power as their workload increases for cooling the environment.

In summary, it could be concluded that although server virtualization technologies provide a method of reducing physical space, carbon footprint and most importantly electrical costs, overall Green IT and cost savings could be achieved through a combination of IT equipment and Site infrastructure.

## Figures and Tables

**Table 1.** Processing workloads.

**Workload 1**	**Idle** – The physical/virtual system is fully booted up and only performing housekeeping tasks.
**Workload 2**	**Light** – The physical/virtual system's RAM, NIC, CPU and DISK are undergoing light processing
**Workload 3**	**Medium** – The physical/virtual system's RAM, NIC, CPU and DISK are undergoing medium processing
**Workload 4**	**Heavy** – The physical/virtual system's RAM, NIC, CPU and DISK are undergoing heavy processing

WORKLOAD 1 = 10 – 15 % Utilization

WORKLOAD 2 = 20 – 25 % Utilization

WORKLOAD 3 = 25 – 30 % Utilization

**Table 2.** Comparison between the consumption of virtualized & physical servers.

	**Virtualized**	**Physical**

**No of Clients**	**2 VMs**	**2 servers**
**Idle**	104.5	207.6
**Workload 1**	111.4	209.1
**Workload 2**	117	230.2
**Workload 3**	121.4	234.9
